# Specific shifts in the endocannabinoid system in hibernating brown bears

**DOI:** 10.1186/s12983-020-00380-y

**Published:** 2020-11-23

**Authors:** Christian Boyer, Laura Cussonneau, Charlotte Brun, Christiane Deval, Jean-Paul Pais de Barros, Stéphanie Chanon, Nathalie Bernoud-Hubac, Patricia Daira, Alina L. Evans, Jon M. Arnemo, Jon E. Swenson, Guillemette Gauquelin-Koch, Chantal Simon, Stéphane Blanc, Lydie Combaret, Fabrice Bertile, Etienne Lefai

**Affiliations:** 1 Université Clermont Auvergne, INRAE, UNH, Clermont-Ferrand, France; 2 Université de Strasbourg, CNRS, IPHC UMR 7178, Strasbourg, France; 3 Plateforme de Lipidomique, INSERM UMR1231, Université de Bourgogne, Dijon, France; 4 Université de Lyon, INSERM, INRAE, INSA, Functional Lipidomic Plateform, Lyon, France; 5 Department of Forestry and Wildlife Management, Inland Norway University of Applied Sciences, Campus Evenstad, NO-2480 Koppang, Norway; 6 Department of Wildlife, Fish, and Environmental Studies, Swedish University of Agricultural Sciences, SE-901 83 Umeå, Sweden; 7 Faculty of Environmental Sciences and Natural Resource Management, Norwegian University of Life Sciences, NO-1432 Ås, Norway; 8 Centre National d’Etudes Spatiales, CNES, F-75001 Paris, France

**Keywords:** Hibernation, Brown bear, Metabolism, Lipidomic, Docosahexaenoic acid, Endocannabinoid system, Cannabinoid receptor 1, Cannabinoid receptor 2, 2-arachidonoylglycerol, Anandamide, N-oleoylethanolamide

## Abstract

In small hibernators, global downregulation of the endocannabinoid system (ECS), which is involved in modulating neuronal signaling, feeding behavior, energy metabolism, and circannual rhythms, has been reported to possibly drive physiological adaptation to the hibernating state. In hibernating brown bears (*Ursus arctos*), we hypothesized that beyond an overall suppression of the ECS, seasonal shift in endocannabinoids compounds could be linked to bear’s peculiar features that include hibernation without arousal episodes and capacity to react to external disturbance. We explored circulating lipids in serum and the ECS in plasma and metabolically active tissues in free-ranging subadult Scandinavian brown bears when both active and hibernating. In winter bear serum, in addition to a 2-fold increase in total fatty acid concentration, we found significant changes in relative proportions of circulating fatty acids, such as a 2-fold increase in docosahexaenoic acid C22:6 n-3 and a decrease in arachidonic acid C20:4 n-6. In adipose and muscle tissues of hibernating bears, we found significant lower concentrations of 2-arachidonoylglycerol (2-AG), a major ligand of cannabinoid receptors 1 (CB1) and 2 (CB2). Lower mRNA level for genes encoding CB1 and CB2 were also found in winter muscle and adipose tissue, respectively. The observed reduction in ECS tone may promote fatty acid mobilization from body fat stores, and favor carbohydrate metabolism in skeletal muscle of hibernating bears. Additionally, high circulating level of the endocannabinoid-like compound N-oleoylethanolamide (OEA) in winter could favor lipolysis and fatty acid oxidation in peripheral tissues. We also speculated on a role of OEA in the conservation of an anorexigenic signal and in the maintenance of torpor during hibernation, while sustaining the capacity of bears to sense stimuli from the environment.

## Background

To deal with seasonal cold and food shortage during winter, hibernating mammals show a combination of behavioral and physiological changes. To save energy during hibernation, hibernating animals use periods of torpor characterized by decreased metabolic rate and body temperature, reduction in respiratory and heart rates, and physical inactivity [1, 2]. Brown bears (*Ursus arctos*) exhibit unique features, as they hibernate at mild hypothermia (32–35 °C) and can stay inside their dens for up to 7 months, without drinking, eating, defecating or urinating, and with no arousal episodes [3–6]. While denning, they reduce their metabolic rate by about 75% [7], and rely primarily on mobilization of fat stores, which is reflected by increased circulating fatty acid concentration and body fat store depletion during winter [8–10].

Beyond energy substrates, lipids also have pleiotropic actions in the regulation of metabolism, and changes in membrane fatty acid composition have already been described in hibernating animals [11–14], including the brown bear [9]. Membrane phospholipids can also provide long-chain fatty acids for the synthesis of bioactive lipid mediators, such as endocannabinoids [15–17]. The endocannabinoid system (ECS) was originally described as being composed of G-protein coupled receptors (CB1 and CB2) and their endogenous ligands, of which the main ones are derived from arachidonic acid 20:4n-6 (AA) esterified into phospholipids, and called 2-arachidonoyl glycerol (2-AG) and anandamide (AEA) [15–20]. These two well-characterized compounds clearly show varying affinity for CB1 and CB2 receptors. Indeed, AEA is considered as a high affinity CB1-partial agonist (and weak CB2 agonist), whereas 2-AG is described as a low-to-moderate affinity CB1 and CB2 full agonist [21, 22]. 2-AG and AEA belong to the large family of 2-acylglycerols (2-AcGs) and N-acylethanolamines (NAEs), respectively [17, 19]. N-acyl-phosphatidylethanolamine-hydrolyzing phospholipase D (NAPEPLD) and sn-1-specific diacylglycerol lipase-α and β (DAGLA and DAGLB) are the main enzymes involved in the biosynthesis of NAEs and 2-AcGs, respectively [17, 19]. Fatty acid amide hydrolase (FAAH) is responsible for NAEs catabolism (and to a lesser extend for 2-AG) [23], and monoacylglycerol lipase (MGLL) specifically catabolizes 2-AcGs [17, 19]. eCBs can also be metabolized by lipoxygenases (LOXs) and by cyclooxygenase-2 (COX-2), an alternative pathway for eCBs catabolism [17].

The ECS includes structurally related compounds like N-oleoylethanolamine (OEA), called «endocannabinoids-like compounds» (eCBs-like). The latter are metabolized by the same biosynthetic and catabolic enzymes as eCBs [17]. Although eCBs-like compounds are not able to bind to CB1 and CB2 receptors, they can bind to other G-protein coupled receptors (e.g. GPR119 and GPR55) or nuclear receptors, like peroxisome proliferator-activated receptor α (PPARA) [17].

Endogenous cannabinoids are involved in the regulation of many physiological processes, including neuronal signaling [24], stress response [25], metabolism [25–27], feeding behavior and energy storage [25, 28]. Evidences support the fact that the ECS could be involved in sleep cycles [29], circadian and potentially circannual rhythms [30]. At the central level (e.g. hypothalamus), CB1 is able to promote food intake and reduce energy expenditure [25, 31]. In addition, CB1 activation in adipose tissue leads to fatty acid and glucose uptake, and to upregulation of lipogenesis [25]. In liver, CB1 signaling leads to increased expression of genes involved in the synthesis of fatty acids [32], and in skeletal muscle tissue, CB1 activation triggers a decrease in glucose uptake and insulin sensitivity [25]. The CB2 receptor is well known to be widespread over immune cells and to have numerous immunomodulatory roles [33]. CB2 has also been detected in metabolic tissues, like adipose tissue and skeletal muscle [34, 35] and CB2 pharmacological or genetic inactivation in murine obesity models promote insulin-mediated glucose uptake in skeletal muscles, reduce adipose tissue inflammation, and thus improves insulin sensitivity [36, 37]. Finally, the eCB-like OEA promotes lipolysis, fatty acid oxidation in skeletal muscle and liver, and triggers an anorexigenic signal, notably through the nuclear receptor PPARA [38, 39]. Considering the pleiotropic roles of ECS in neuronal signaling, regulation of feeding behavior, energy metabolism and circannual rhythms, important changes are expected during hibernation. Several ECS circulating compounds have been quantified in hibernating black bears, during and around the torpor phase [40],with no major changes observed except a slight increase in 2-AG in the period of metabolic drop before torpor. Although a decrease in ECS tone has been observed in hibernating marmots (*Marmota monax and flaviventris*) and ground squirrels (*Spermophilus richardsonii*) [30, 41, 42], we hypothesize that a similar decrease should occur in hibernating bears, not excluding specific changes due to their unique features during hibernation (mild hypothermia, no periodic arousal, and maintenance of alertness). Therefore, we explored here seasonal variations in fatty acid composition and ECS tone, in both circulating compartment and in muscle and adipose tissues, in winter-hibernating and summer-active brown bears.

## Results

### Seasonal differences in serum lipids

**Fig. 1:**
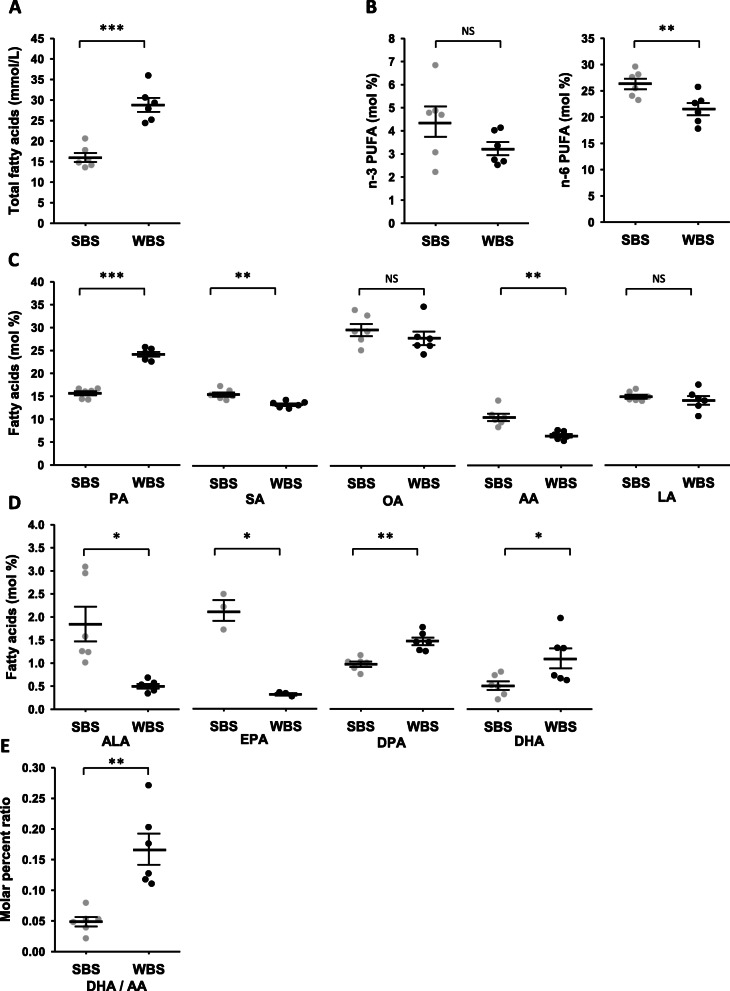
Lipidomic from summer and winter brown bear serum. The winter and summer bear serum mixes were prepared as described (Supplementary Table S1)**. a**: Total fatty acid (FA) concentration. **b**: Total n-6 and n-3 FA relative proportions of total lipids. **c**: Highest molar percent lipid species D: Molar percent of the n-3 family lipid species. **e**: Molar ratios of DHA/AA in summer and winter serum. Detailed lipidomic results are presented in Supplementary Tables S2 and S3. Data are expressed in mmol/L for total FAs concentration, or molar percentage of total lipids and are represented as mean ± SEM of separate extractions and quantifications from the twelve mixes (six summer and six winter serum mixes, except for EPA with data from only three summer and three winter mixes). Paired Student t-test were used to compare wummer and winter data and Benjamini-Hochberg correction was applied for multiple comparisons. * indicates BH adjusted *p* value < 0.05 when comparing seasons, ** for *p* < 0.01, *** for *p* < 0.001, NS: non significant. AA:arachidonic acid, ALA: alpha-linolenic acid, DHA: docosahexaenoic acid, DPA: docosapentaenoic acid, EPA: eicosapentaenoic acid, LA: linoleic acid, OA: oleic acid, PA: palmitic acid, SA: stearic acid, SBS: summer bear serum, WBS: winter bear serum

We explored the fatty acid (FA) composition of winter-hibernating (WBS) and summer-active (SBS) bear serum (see supplementary Table S1). From the lipidomic data, we compared both the summer and winter concentrations and proportions of fatty acids (see supplementary Table S2 and S3 for detailed lipidomic results). As shown in Fig. 1a, the total concentration of FAs was about twofold higher in WBS relative to SBS (28.82 ± 1.71 vs. 15.99 ± 1.09 mmol/L). All but two quantified lipid species were higher in concentration in hibernating bears, i.e. saturated fatty acids (SFAs), monounsaturated fatty acids (MUFAs), and n-6 polyunsaturated fatty acids (PUFAs) (Supplementary Table S2). Only concentrations of alpha-linolenic acid C18:3 n-3 (ALA) (0.49 -fold, non-significant) and eicosapentaenoic acid C20:5 n-3 (EPA) (0.26-fold) were lower in WBS (Supplementary Table S2).

Meanwhile, the molar percent of total n-6 species were found to be lower in WBS compared to SBS (Fig. 1b). Lipid species with the highest molar percent are presented in Fig. 1c (see Supplementary Table S3). Among SFAs, palmitic acid C16:0 (PA) was found in higher proportion, whereas stearic acid C18:0 (SA) was in lower proportion in winter serum. Similar proportions of oleic acid 18:1n-9 (OA), belonging to the n-9 MUFAs, were found in winter and summer bear serum. Concerning n-6 PUFAs, the proportion of arachidonic acid C20:4 n-6 (AA) was lower during winter, whereas proportion of linoleic acid C18:2 n-6 (LA) remained unchanged (Fig. 1c). For individual species of the n-3 family (Fig. 1d and Supplementary Table S3), docosapentaenoic acid C22:5 n-3 (DPA, 1.5-fold) and docosahexaenoic acid C22:6 n-3 (DHA, 2.2-fold) were found in higher proportions.

The proportion of C20:5 n-3 (EPA) was found much lower (0.15-fold) in winter serum, as well as the alpha-linolenic acid C18:3 n-3 (ALA, 0.27-fold), a precursor of the EPA, DPA and DHA species.

From molar percent values, the DHA/AA ratio was 3.2-fold higher in winter (Fig. 1e).

### Changes in plasma endocannabinoids and endocannabinoids-like compounds

**Fig. 2:**
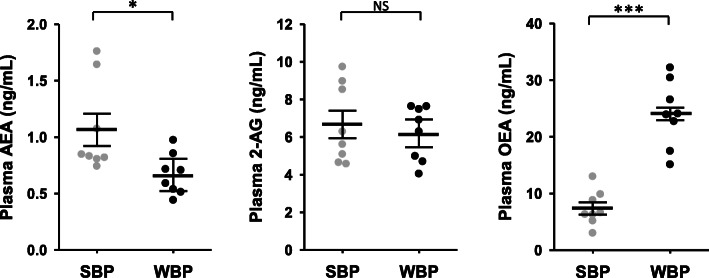
Circulating endocannabinoids concentration in brown bear plasma. Concentration of three major endocannabinoids compounds in bear plasma. Plasma were collected from bears at both winter-hibernating and summer-active time points (Supplementary Table S1). Data are expressed in ng/mL and are represented as mean ± SEM of separate extractions and quantifications (*n* = 8). Paired Student t-test were used to compare wummer and winter data. * indicates p value < 0.05 when comparing seasons, *** for *p* < 0.001, NS: non significant. AEA: anandamide, 2-AG: 2-arachidonoylglycérol, eCBs: endocannabinoids, OEA: N-oleoylethanolamine, SBP: summer bear plasma, WBP: winter bear plasma

We next assessed circulating eCBs and eCBs-like in bear plasma. Paired samples were collected in winter and in summer from eight bears (Supplementary Table S1) and quantification of AEA, 2-AG and OEA are presented in Fig. 2 and supplementary Table S5. Lower concentrations were observed for AEA (0.63-fold) in winter compared to summer, whereas the reverse was observed for OEA (3.3-fold). No difference was found for 2-AG plasma concentration.

### Changes in endocannabinoid concentrations in muscle and adipose tissues

**Fig. 3:**
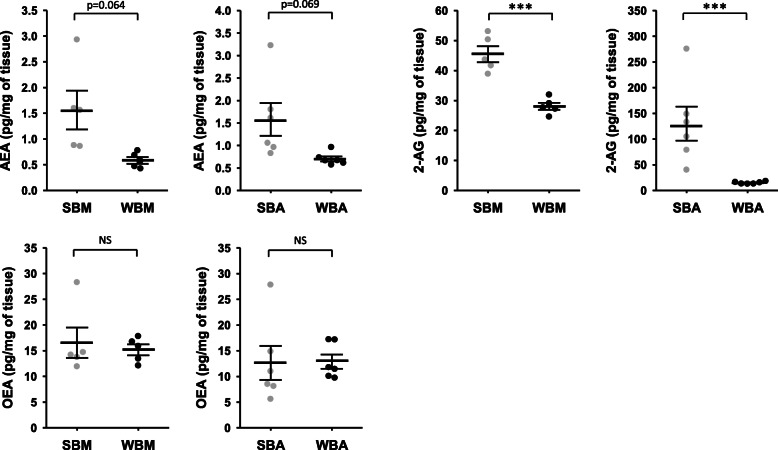
Endocannabinoids concentration in brown bear muscle and adipose tissue. Concentration of three major endocannabinoids compounds in bear muscle and adipose tissue. Tissues were collected from bears at both winter-hibernating and summer-active time points (Supplementary Table S1). Data are expressed in pg/mg and are represented as mean ± SEM of separate extractions and quantifications (*n* = 5 for muscle tissue and *n* = 6 for adipose tissue). Paired Student t-test were used to compare wummer and winter data. *** indicates p value < 0.0001 when comparing seasons, NS: non significant. AEA: anandamide, 2-AG: 2-arachidonoylglycérol, OEA: N-oleoylethanolamine, SBA: summer bear adipose tissue, SBM: summer bear muscle, WBA: winter bear adipose tissue, WBM: winter bear muscle

Quantification of endocannabinoids was then performed in bear muscle and adipose tissues. Paired tissues samples were collected from bears in winter and in summer (Supplementary Table S1) and quantification of AEA, 2-AG and OEA are presented in Fig. 3 and supplementary Table S5. AEA concentration was lower in both muscle and adipose tissues during winter versus summer, close to the statistical threshold (*p* = 0.064 and *p* = 0.069, respectively). 2-AG concentration was significantly lower in muscle and adipose tissues samples during winter, by about 1.6- and 9-fold, respectively. By contrast, no seasonal changes were found in OEA concentrations in both muscle and adipose tissues.

### Changes in endocannabinoid pathway-related gene expressions in muscle and adipose tissues

**Fig. 4:**
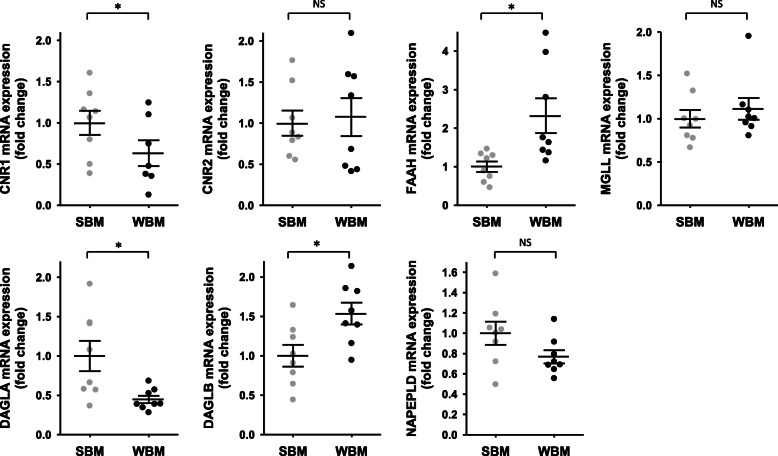
Fold change in gene expression of target genes involved in endocannabinoids biosynthesis and catabolism in brown bear muscle tissue. Muscle tissues were collected from bears at both winter-hibernating and summer-active time points (Supplementary Table S1), total RNA was extracted and expression levels were measured by RT-qPCR. Data are normalized against TBP mRNA levels and expressed as a fold change relative to the summer condition, represented as mean ± SEM of separate extractions and quantifications (*n* = 8). Paired Student t-test were used to compare wummer and winter data. * indicates p value < 0.05 when comparing seasons, NS: non significant. CNR1: cannabinoid receptor 1, CNR2: cannabinoid receptor 2, DAGLA: diacylglycerol lipase alpha, DAGLB: diacylglycerol lipase beta, FAAH: fatty acid amide hydrolase, MGLL: monoacylglycerol lipase, NAPEPLD: N-acyl phosphatidylethanolamine phospholipase D, SBM: summer bear muscle, WBM: winter bear muscle

To explore tissue metabolism of endocannabinoids, we quantified gene expression in muscle and adipose tissue of the eCBs membrane receptors CB1 and CB2, and several enzymes involed in the synthesis and catabolism of eCBs. For muscle tissue, paired samples were from 8 bears at the two time points, while for adipose tissue, data are coming from 5 bears in summer and 13 bears in winter (Supplementary Table S1). Data are presented in Fig. 4 and Supplementary Table S5.

For genes that encode the membrane receptors CB1 and CB2 in muscle tissue, CNR1 mRNA level, but not CNR2, was decreased (0.63-fold) in winter (Fig. 4). Concerning enzymes that catabolize AEA and 2-AG, mRNA level of FAAH was induced (2.3-fold) in winter, but MGLL gene expression did not change. For genes encoding enzymes of the biosynthetic pathway, DAGLA mRNA level was strongly reduced in muscle tissue during winter (0.40-fold), whereas DAGLB mRNA level was increased (1.53-fold). Finally, gene expression of NAPEPLD did not change in muscle (Fig. 4).

**Fig. 5:**
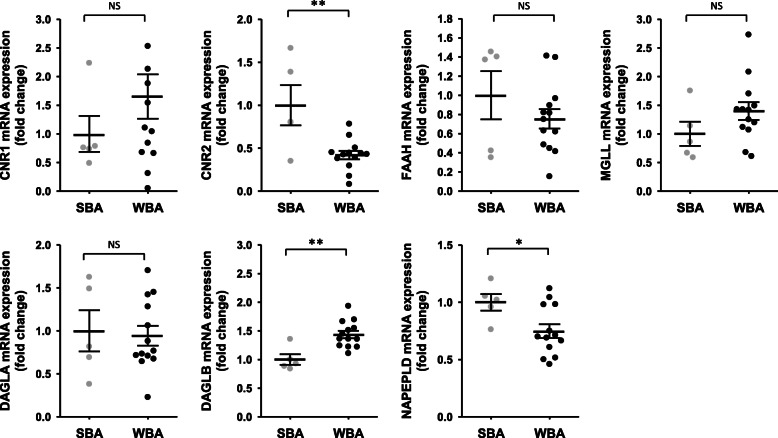
Fold change in gene expression of target genes involved in endocannabinoids biosynthesis and catabolism in brown bear adipose tissue. Adipose tissues were collected from bears at both winter-hibernating and summer-active time points (Supplementary Table S1), total RNA was extracted and expression levels were measured by RT-qPCR. Data are normalized against TBP mRNA levels and expressed as a fold change relative to the summer condition, represented as mean ± SEM of separate extractions and quantifications (*n* = 5 for summer and *n* = 13 for winter samples). Unpaired Student t-test were used to compare wummer and winter data.* indicates *p* value < 0.05 when comparing seasons. CNR1: cannabinoid receptor 1, CNR2: cannabinoid receptor 2, DAGLA: diacylglycerol lipase alpha, DAGLB: diacylglycerol lipase beta, FAAH: fatty acid amide hydrolase, MGLL: monoacylglycerol lipase, NAPEPLD: N-acyl phosphatidylethanolamine phospholipase D, SBA: summer bear adipose tissue, WBA: winter bear adipose tissue

Conversely, in adipose tissue (Fig. 5), no significant changes in CNR1 gene expression were reported whereas CNR2 expression was strongly decreased in winter (0.42-fold). For gene expression of catabolic enzymes (FAAH and MGLL), did not change in adipose tissue between seasons. Finally, for genes encoding biosynthetic enzymes, mRNA levels of DAGLB and NAPELD were respectively found higher (1.44-fold) and lower (0.75-fold) in winter.

## Discussion

Thanks to repeated capture sessions, we were able to gather samples of serum, plasma and tissues from high number of free-living brown bears (*Ursus arctos*). From the 28 bears included in this study, samples were collected both in February during winter hibernation and in June during summer active period. Due to limited amount of available biological material, the analyses were performed on samples coming from different subsets of the 28 bears. In all but adipose tissue, analyses were performed on winter and summer paired samples (Supplementary Table S1). We examined circulating lipid and ECS compounds in both summer-active and winter-hibernating brown bears to explore the extent to which regulation of the ECS reflects bear hibernation peculiarities, including survival due to lipid oxidation, maintenance of muscle glycolysis, and maintained alertness during dormancy. The seasonal shift we highlighted in serum FAs composition, together with a decrease in tissue AEA and 2-AG, and a three-fold increase in circulating OEA during winter, could contribute to the behavioral and metabolic changes that occur in hibernating bears.

Hibernators experience extended periods of food shortage during hibernation and primarily rely on mobilization of fat stores from white adipose tissue [1]. Accordingly, we found that the concentration of total circulating fatty acids was elevated in hibernating bears, a finding in line with previous studies [5, 43]. Considering both the amount and relative proportions of circulating lipids, our results are consistent with changes in serum and plasma lipid profiles during hibernation that have been previously published [5, 9, 10], notably an enrichment in DHA C22:6 n-3 and depletions in ALA C18:3 n-3 and EPA C20:5 n-3, during winter compared to summer. Whether the depletion in the ALA and EPA precursor species could be directly linked to the observed DHA increase remains to be elucidated.

Here, the DHA serum enrichment that we observed in hibernating bears is actually not coming from dietary FAs intake but rather due to lipid stores mobilization. The health benefits that have been attributed to n-3 PUFAs (e.g. DHA), essentially triggered by DHA dietary intervention studies, could potentially be transposed in the context of hibernation. Indeed, it has already been hypothesized that DHA could be involved in the bear’s resistance to muscle atrophy during hibernation [10]. DHA appears to prevent muscle atrophy in fasting mice, and increases muscle glycogen stores [44]. Strikingly, in parallel to DHA serum enrichment, hibernating bears have more than a 3-fold higher glycogen muscle content compared to summer-active animals [10]. In addition to its anti-inflammatory effects, DHA is also known to exert a positive effect on protein balance by decreasing expression of factors involved in protein breakdown [45] and enhancing protein synthesis, notably by promoting mammalian Target Of Rapamycin (mTOR) activation [46].

Concomitantly to serum DHA enrichment, we observed a drop in AA proportion, thus leading to a sharp increase in the DHA/AA ratio. Omega-3/omega-6 ratio is known to have an impact on global health [47], and the balance of this ratio could also impact the endocannabinoid system [48], notably because AA is a precursor of the two main eCBs 2-AG and AEA. Indeed, n-6 PUFAs-enriched diets have been shown to increase the level of 2-AG or AEA in the brain, plasma, and peripheral tissues in non-hibernating animal models [49–52]. It is noteworthy to mention that, in response to DHA supplementation, an enrichment of this fatty acid in phospholipids of cell membranes occurs in parallel with a decrease in AA content [38, 49, 53, 54]. By remodeling the amount of AA-containing phospholipids, DHA is able to reduce the synthesis of AEA and 2-AG [49, 54]. Further studies on bears, focusing on fatty acid membrane composition in tissues at different time points, will be helpful to characterize the remodeling of membrane lipids that could affect the availability of FAs precursors for eCBs biosynthesis. Data on eCBs compounds from experimental short fasting in non-hibernating mammals are very divergent, depending on the tissue considered (e.g. brain or peripheral tissues) and the duration of food deprivation, but tissue levels of eCBs are mainly regulated by the availability of their membrane phospholipid precursors and by the activity of biosynthetic and catabolic enzymes [28, 49, 55, 56].

We hypothesized that drastic reduction in metabolic activity, lack of intake of dietary PUFAs, significant increase in the serum DHA/AA ratio, and perhaps reduction in tissue AA-phospholipids concentration, could lead to a global reduction in ECS tone during the hibernation period. The reduction in ECS tone has already been documented in hibernating marmots [30, 41], but not confirmed in large-bodied hibernators.

Comparing active and hibernation states in brown bears, we reported here a decrease in plasma concentration of AEA, and an unexpected 3-fold increase in OEA circulating levels in hibernating bears. In both muscle and adipose tissues, 2-AG and AEA (close to statistical threshold) were found lower in winter, while OEA did not change. Quantification of winter serum eCBs was previously reported in black bears during and around the topor phase, but summer active bears were not investigated [40]. Nutritional status of the captured animals and diet were not specified. These elements strongly limit comparison between the two studies.

Taken together, our data allowed us to make several hypotheses about possible mechanisms by which ECS could contribute to the metabolic and behavioral changes that occur in bears during hibernation. First, considering that AEA and 2-AG CB1 agonists favor food intake and stimulate lipogenesis [25], CB1 signaling is expected to be upregulated during the active summer period in order to promote energy storage, and downregulated during winter hibernation to stimulate lipolysis and FAs oxidation. The tissue concentration drops in 2-AG and AEA observed during winter could be due to a decrease in tissue AA-phospholipids concentration, as we hypothesized above. The degradation of AEA could also be increased in muscle tissue during hibernation, as reflected in the higher mRNA levels of FAAH, the main hydrolase that degrades AEA [19, 23]. In adipose tissue, lower NAPEPLD mRNA level content during hibernation may support a decrease in AEA synthesis, and ultimately content. The tissue content in 2-AG is decreased in winter with no changes in mRNA levels of the catabolic enzyme MGLL. Furthermore, opposite changes in DAGLA and DAGLB gene expression do not allow to speculate on the biosynthetic/degradation balance. One limitation of our study is that gene expression could not reflect biological activity. Moreover, we only focused on main biosynthetic and catabolic enzymes involved in eCBs metabolism, and investigation on alternative degradation route as endocannabinoid oxygenation by cyclooxygenases and lipoxygenases would bring new insights.

During hibernation, lower 2-AG (and AEA close to statistical threshold) tissue content and the reduction of CNR1 and CNR2 mRNA levels in muscle and adipose tissue, respectively, strongly support reduced ECS tone in both tissues. In non-hibernating mammals, pharmacological inhibition of CB1 leads to a decrease in PDK4 expression [25, 57]. PDK4 is a major negative regulator of PDH activity, that in turn regulates the whole body oxidative carbohydrate metabolism. In hibernating bear muscle, recent studies have shown that PDK4 is upregulated compared to summer active state [10, 58] and expression of PDK4 during hibernation appear thus to be disconnected from direct regulation by CB1. CB1 receptor antagonism also leads to an increased uptake of glucose in muscle via PI3K signaling [59], and glycolysis appears preserved in bear skeletal muscle during hibernation, as suggested by an overall increase in the protein abundance of all glycolytic enzymes [10]. As proposed by Chazarin et al. and Vella et al., bears still oxidize glucose and produce lactate in skeletal muscle during hibernation [10, 60].

Overactivation of the ECS is a hallmark of obesity [61, 62], and 2-AG is predominantly found in higher concentration in tissues of obese people [61, 63]. Interestingly, in murine models of obesity, gain of adipose tissue often leads to increased fat inflammation [36, 37]. Genetic or pharmacological inactivation of CB2 receptor contribute to reduce adipose tissue inflammation, increase insulin sensitivity and skeletal muscle glucose uptake [36, 37]. Strikingly, insulin resistance has been described in hibernating bears adipocytes [64]. As bears don’t experience health consequences of circannual high body fat storage [65], a reduced CB2 signaling in adipose tissue could dampen adipose tissue inflammation. Lower amounts of 2-AG and AEA could also reduced CB1 signaling in adipose tissue, thus limiting lipogenesis and promoting lipolysis during hibernation in bears, as also suggested for hibernating marmots [30].

OEA is a high-affinity agonist for peroxisome proliferator-activated receptor α (PPARA), regulating food intake and stimulating fat catabolism [38, 39, 53, 66, 67]. The eCB-like OEA is generally synthesized in response to dietary oleic acid intake by enterocytes of the small intestine [49, 54], and inhibits food intake. It has already been shown in rodents that food deprivation inhibits OEA synthesis in the small intestine, but stimulates its synthesis in liver [38, 53, 68, 69]. Therefore, during bear hibernation, circulating OEA could originate from tissue synthesis (probably hepatic) and be released in the blood flow. The high OEA level that we found in hibernating bears, not triggered by food intake, could participate in a sustained anorexigenic signal during the hibernation state.

Consequences of high levels of circulating OEA have been studied in non-hibernating rodents. Intraperitoneal OEA administration in rats notably impairs locomotor activity, which is supported by a decrease in ambulation, an increase of the time spent in inactivity, and the presence of signs of catalepsy [66, 70]. We thus can hypothesize that a higher amount of plasma OEA during bear hibernation can participate in the maintenance of prolonged physical inactivity. It has also been shown that intracerebroventricular injections of OEA promote alertness, with the observation of enhanced dopamine and c-Fos expressions in wake-related brain areas [71]. Bears are known to stay sensitive to disturbance during hibernation [72–74]. High circulating amounts of OEA might thus participate in alertness to external stimuli from the environment in hibernating bears. OEA during winter possibly also favors body fat mobilization for energy needs, with stimulation of FA and glycerol release from adipocytes [38, 39]. Finally, a potential role for OEA in the promotion of fasting-induced ketogenesis during hibernation could also be considered, as OEA has been demonstrated to increase 3-hydroxybutyrate production in in vivo rodent models [38, 39].

## Conclusions

**Fig. 6:**
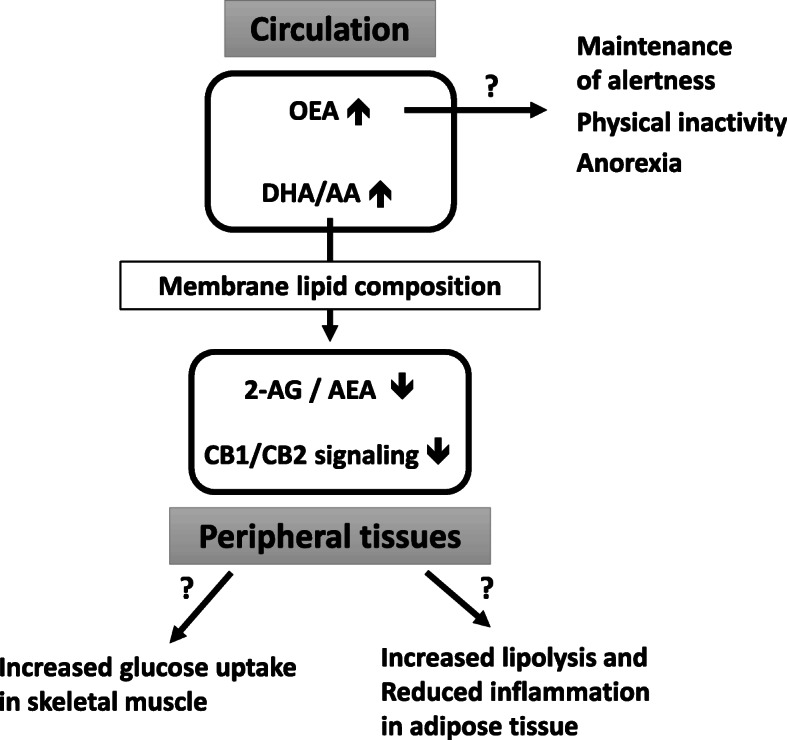
Hypothetical consequences of changes in circulating lipids and endocannabinoid system tone during hibernation in brown bear. Black arrows represent possible behavior and metabolic outcomes

In conclusion, our results show a reduction in ECS tone in hibernating bears and suggest a coordinated downregulation of CB1 and CB2 signaling in skeletal muscle and adipose tissue. As summarized in Fig. 6, these features could favor energy mobilization through lipolysis, and optimization of glucose uptake by skeletal muscles. Despite high fat stores in winter, bears do not exhibit features of ECS overactivation, and decrease in CB2 signaling could dampen adipose tissue inflammation. The observed increase in circulating OEA level may participate in the behavioral and physiological adaptations during bear hibernation state, like maintenance of an anorexigenic signaling pathway, and promotion of lipolysis and fatty acid β-oxidation. We also speculated about OEA involvement in torpor maintenance and in motor activity reduction, as well as a role in conservation of alertness at the level of central nervous system.

## Methods

### Bear sample collection

A total of 28 free ranging subadult brown bears (*Ursus arctos*) from Dalarna and Gävleborg counties, Sweden, were included in this study, including 4 bears captured two consecutive years. All samples and data were collected under protocols approved by the Swedish Ethical Committee on Animal Experiment (applications Dnr C3/2016 and Dnr C18/2015), the Swedish Environmental Protection Agency (NV-00741-18), and the Swedish Board of Agriculture (Dnr 5.2.18–3060/17). All procedures complied with Swedish laws and regulations.

As described previously [10, 75], blood, subcutaneous adipose tissue, and muscle tissue (vastus lateralis) samples were collected at two time points, in February during winter hibernation (W) and in June during summer-active period (S). Blood samples were collected from the jugular vein into 8 ml dry tubes for serum (Vacuette® Z serum Sep Clot Activator, Greiner Bio-One GmbH, Kremsmünster, Austria) or into 10 ml EDTA-coated tubes (BD Vacutainer®, FisherScientific, Illkirch, France) for plasma.

The analyses were performed on samples coming from different subsets of bears as described in Supplementary Table S1.

### Lipid extraction and analysis

To perform serum lipidomic analysis, serum mixes were prepared as followed: for a given year, 50 μl of summer serum from each bear of the year was pooled to obtain the summer mix. In parallel, 50 μl of winter serum from the same bears was pooled to obtain the winter mix. A total of 6 summer and winter paired mixes were obtained (Supplementary Table S1). Lipids were extracted and analyzed as previously described [76]. After addition of an internal standard (tri-17:0 triacylglycerol), total lipids were extracted twice from bear serum mixes with ethanol/chloroform (1:2, v/v). The organic phases were dried under nitrogen and lipids were transmethylated. Briefly, samples were treated with toluene-methanol (1:1, v/v) and boron trifluoride in methanol (14%). Transmethylation was carried out at 100 °C for 90 min in screw-capped tubes. Then 1.5 mL K_2_CO_3_ in 10% water was added and the resulting fatty acid methyl esters were extracted by 2 mL of isooctane and analyzed by gas chromatography (GC) with a HP6890 instrument equipped with a fused silica capillary BPX70 SGE column (60 × 0.25 mm). The vector gas was hydrogen. Temperatures of the Ross injector and the flame ionization detector were set to 230 °C and 250 °C, respectively. Data were expressed in mmol/L for total or individual fatty acids (FAs) concentration or molar percentage of total lipids for individual FAs. Detailed lipidomic results are presented in supplementary Table S2 (serum fatty acid concentrations) and S3 (serum fatty acid relative proportions).

### Endocannabinoid quantification

For quantification of circulating endocannabinoids, analysis was performed on 500 μl of plasma collected at the two time points (S and W) from 8 individual animals (see supplementary Table S1). Standard endocannabinoids (eCBs), i.e.- PEA, PEA-d5, OEA, OEA-d4, AEA, AEA-d4, 2AG, and 2AG-d5, were purchased from Cayman (Bertin BioReagent, Saint-Quentin en Yvelines, France). Mass spectrometry quality grade solvents were purchased from Fischer Scientific (Illkirch, France). Tissue samples (adipose and muscle tissues); c.a 100 mg) were crushed in an Omni Bead Ruptor 24 apparatus (Omni International, Kennesaw, USA) with circa twenty 1.4 mm OD zirconium oxide beads (S = 6.95 m/s, T = 30s, C = 3; D = 10s) and 900 μl of methanol/Tris-buffer (50 mM, pH = 8) 1/1 containing 20 ng of PEA-d5, 2 ng OEA-d4, 10 ng AEA-d4, and 20 ng 2AG-d5. Then, each homogenate was added with 2 mL of CHCl_3_/MeOH (1:1, v/v) and 500 μL of Tris (50 mM, pH = 8), vortexed and centrifuged 10 min at 3000 g. The organic layer was recovered and the upper aqueous phase was extracted twice with chloroform (1 mL). Finally, organic phases were pooled and evaporated under vacuum.

Plasma (500 μL) were mixed with 500 μL cold methanol containing 11 ng AEA. After protein precipitation at − 20 °C for 2 h, endocannabinoids were extracted with methanol/chloroform (1:1, v/v) (5 ml) and saline (1.25 mL). The organic phase was recovered and the aqueous phase was extracted twice with chloroform (3 mL). Organic phases were finally pooled and evaporated under vacuum.

Dried extracts were solubilized with methanol (200 μL) and centrifuged for 5 min at 20,000 g. Four microliters of the supernatant were injected into a 1200 LC system coupled to a 6460-QqQ MS/MS system equipped with an ESI source (Agilent technologies). Separation was achieved on Zorbax SB-C18 2.1 × 50 mm, 1.8 μm column (Agilent technologies) at a flow rate of 0.4 mL/min, 40 °C, with a linear gradient of (solvent A) water containing 0.1% formic acid and (solvent B) methanol containing 0,1% formic acid as follows: 10% of B for 1 min, up to 85% of B in 8 min, and then 100% B for 4.5 min. Acquisition was performed in positive Selected Reaction Monitoring (SRM) mode (source temperature: 350 °C, nebulizer gas flow rate: 10 L/min, 40 psi, sheath gas flow 10 L/min, sheath gas temperature 350 °C, capillary 4000 V, nozzle 1000 V).

Transitions used were: 2AG-d5 384.3 → 91.1 (frag 120 V, CE 62 V), 2AG 379.1 → 91 (frag 120 V, CE 62 V), AEA-d4 352.2 → 66.1 (frag 115 V, CE 14 V), AEA 348.2 → 62 (frag 120 V, CE 14 V), OEA-d4 330.2 → 66.1 (frag 120 V, CE 14 V), OEA 326.2 → 62 (frag 115 V, CE 14 V), PEA-d5 305.2 → 62 (frag 124 V, CE 14 V), and PEA 300.2 → 62 (frag 124 V, CE 14 V).

Endocannabinoids quantification in tissues was performed on tissue samples collected at the two time points (S and W) from 5 (muscle tissue) and 6 (adipose tissue) bears (Supplementary Table S1). eCBs from tissues were quantitated according to the isotope dilution method. Results are expressed as pg per mg of wet weight of tissue. eCBs from plasma were quantitated using calibration curves obtained with authentic standards extracted by the same method used for plasma samples. Linear regression was applied for calculations. Results are expressed as ng of endocannabinoid per mL of plasma.

### Quantification of mRNAs by real-time RT-PCR

For mRNA quantification using RT-qPCR, total RNAs were obtained from muscle and adipose tissues collected at the two time points (S and W). For the muscle tissue, RNAs were extracted from 8 bears in summer and winter, while for adipose tissue, RNAs were extracted from 5 bears in summer and 13 bears in winter (Supplementary Table S1).

Muscle and adipose tissue total RNA was isolated using the TRIzol reagent (Invitrogen, Courtaboeuf, France) according to the manufacturer’s instructions. First-strand cDNAs were synthesized from 1 μg of total RNA using the PrimeScript RT kit (Ozyme, saint quentin en Yveline, France) with a mixture of random hexamers and oligo(dT) primers, and treated with 60 units of RnaseH (Ozyme). Real-time PCR assays were performed with Rotor-Gene 6000 (Qiagen, Courtaboeuf, France). The primers and real-time PCR assay conditions are listed in supplementary Table S4. The results were normalized by using TBP (TATA box binding protein) mRNA concentration, measured as reference gene in each sample.

### Statistical analysis

Statistical analysis was performed using the R software environment v3.0.2 [77]. For each set of values, distribution of the data was tested using the Shapiro-Wilk normality test, and using the *p* = 0.01 threshold normal distribution was considered in all cases. Differences between summer and winter data were tested using paired Student t-test for lipidomic, endocannabinoid quantification in plasma and tissues, and mRNA level in muscle tissue. For mRNA level in adipose tissue, differences between summer and winter data were tested using unpaired Student t-test. For multiple comparison (lipidomic data), the Benjamini-Hochberg correction using the p.adjust function (Package *stats* version 4.0.0 of R studio) was applied. Data are presented as means ± SEM and individual values are plotted as grey and black dots for respectively summer and winter values. Means, SEM, fold change and associated *p*-values are reported in supplementary Tables S2 to S5. Statistical significance was considered with *p* values or adjusted p values lower than 0.05.

## Supplementary Information

**Additional file 1 Table S1.** Characteristics of brown bears included in the study. **Table S2.** Serum fatty acid concentrations (mmol/L) in winter hibernating (WBS) and summer active (SBS) bears. **Table S3.** Serum fatty acid relative proportions (mol %) in winter hibernating (WBS) and summer active (SBS) bears. **Table S4.** List of primers used for RT-qPCR. **Table S5**: Endocannabinoids (eCBs) and mRNA quantification in plasma and tissues in winter hibernating (W) and summer active (S) bears.

## Data Availability

The datasets generated during and/or analyzed during the current study available from the corresponding author on reasonable request.

## Abbreviations

AA: Arachidonic acid
2-AcG: 2-acylglycerol
AEA: Anandamide
2-AG: 2-arachidonoylglycerol
ALA: Alpha-linolenic acid
AMPK: AMP-activated protein kinase
CNR1: Cannabinoid receptor 1 (gene)
CB1: Cannabinoid receptor 1
CNR2: Cannabinoid receptor 2 (gene)
CB2: Cannabinoid receptor 2
DAGLA: Diacylglycerol lipase α
DAGLB: Dicacylglycerol lipase β
DPA: Docosapentaenoic acid
DHA: Docosahexaenoic acid
eCB: Endocannabinoid
eCB-like: Endocannabinoid-like compound
ECS: Endocannabinoid system
EPA: Eicosapentaenoic acid
FA: Fatty acid
FAAH: Fatty acid amide hydrolase
GPR55: G protein-coupled receptor 55
GPR119: G protein-coupled receptor 119
LA: Linoleic acid
MGLL: Monoacylglycerol lipase
mTOR: Mammalian target of rapamycin
MUFA: Monounsaturated fatty acid
NAE: N-acyl-phosphatidylethanolamine
NAPEPLD: N-acyl-phosphatidylethanolamine-hydrolyzing phospholipase D
OA: Oleic acid
OEA: N-oleoylethanolamide
PA: Palmitic acid
PDH: Pyruvate dehydrogenase
PDK4: Pyruvate dehydrogenase kinase 4
PPARA: Peroxisome proliferator-activated receptor α
PUFA: Polyunsaturated fatty acid
SA: Stearic acid
SBA: Summer bear adipose tissue
SBM: Summer bear muscle
SBP: Summer bear plasma
SBS: Summer bear serum
SFA: Saturated fatty acid
WBA: Winter bear adipose tissue
WBM: Winter bear muscle
WBP: Winter bear plasma
WBS: Winter bear serum
