# Supplementary material for: Specific shifts in the endocannabinoid system in hibernating brown bears
Source: Front Zool. 2020 Nov 23;17:35. doi: 10.1186/s12983-020-00380-y (PMC7681968; doi:10.1186/s12983-020-00380-y)
Supplement: Supplementary file 1 — Additional file 1 Table S1. Characteristics of brown bears included in the study. Table S2. Serum fatty acid concentrations (mmol/L) in winter hibernating (WBS) and summer active (SBS) bears. Table S3. Serum fatty acid relative proportions (mol %) in winter hibernating (WBS) and summer active (SBS) bears. Table S4. List of primers used for RT-qPCR. Table S5: Endocannabinoids (eCBs) and mRNA quantification in plasma and tissues in winter hibernating (W) and summer active (S) bears. [file 12983_2020_380_MOESM1_ESM.docx]

**Supplementary Table S1: Characteristics of brown bears included in the study**

| **ID_number** | **Year of collection** | **Age (year)** | **Gender** | **Serum** | | **Plasma** | | **Muscle Tissue** | | **Adipose Tissue** | | **Muscle tissue RNA** | | **Adipose tissue RNA** | |
| --- | --- | --- | --- | --- | --- | --- | --- | --- | --- | --- | --- | --- | --- | --- | --- |
|  |  |  |  | **S** | **W** | **S** | **W** | **S** | **W** | **S** | **W** | **S** | **W** | **S** | **W** |
| w0825 | 2011 | 4 | F |  |  | X | X |  |  |  |  |  |  |  |  |
| w0904 |  | 3 | F |  |  | X | X |  |  |  |  |  |  |  |  |
| w1004 |  | 2 | M |  |  | X | X |  |  |  |  |  |  |  |  |
|  |  |  |  |  |  |  |  |  |  |  |  |  |  |  |  |
| w1011 | 2012 | 3 | F | Mix1 | Mix1 |  |  |  |  |  |  |  |  |  |  |
| w1017 |  | 3 | F |  |  |  |  |  |  |  |  |  |  |  |  |
| w1104 |  | 2 | F |  |  |  |  |  |  |  |  |  |  |  |  |
| w1105 |  | 2 | F |  |  |  |  |  |  |  |  |  |  |  |  |
| w1110 |  | 2 | F |  |  |  |  |  |  |  |  |  |  |  |  |
|  |  |  |  |  |  |  |  |  |  |  |  |  |  |  |  |
| w1104 | 2013 | 3 | F | Mix2 | Mix2 |  |  |  |  |  |  |  |  | X |  |
| w1105 |  | 3 | F |  |  | X | X |  |  |  |  |  |  |  |  |
| w1110 |  | 3 | F |  |  | X | X |  |  |  |  |  |  |  | X |
| w1204 |  | 2 | M |  |  | X | X |  |  |  |  |  |  |  | X |
| w1207 |  | 2 | M |  |  | X | X |  |  |  |  |  |  | X | X |
| w1209 |  | 2 | F |  |  | X | X |  |  |  |  |  |  |  | X |
|  |  |  |  |  |  |  |  |  |  |  |  |  |  |  |  |
| w1303 | 2014 | 2 | F | Mix3 | Mix3 |  |  |  |  |  |  |  |  |  | X |
| w1304 |  | 2 | F |  |  |  |  |  |  |  |  | X | X |  |  |
| w1305 |  | 2 | F |  |  |  |  |  |  |  |  |  |  |  | X |
| w1316 |  | 2 | M |  |  |  |  |  |  |  |  | X | X |  | X |
| w1317 |  | 2 | M |  |  |  |  |  |  | X | X | X | X |  |  |
|  |  |  |  |  |  |  |  |  |  |  |  |  |  |  |  |
| w1404 | 2016 | 3 | M | Mix4 | Mix4 |  |  | X | X | X | X | X | X | X | X |
| w1407 |  | 3 | F |  |  |  |  |  |  | X | X | X | X | X | X |
| w1509 |  | 2 | F |  |  |  |  | X | X |  |  | X | X | X | X |
| w1511 |  | 2 | F |  |  |  |  |  |  |  |  | X | X |  | X |
| w1512 |  | 2 | F |  |  |  |  |  |  |  |  | X | X |  |  |
|  |  |  |  |  |  |  |  |  |  |  |  |  |  |  |  |
| w1509 | 2017 | 3 | F | Mix5 | Mix5 |  |  |  |  |  |  |  |  |  |  |
| w1601 |  | 2 | M |  |  |  |  |  |  | X | X |  |  |  |  |
| w1608 |  | 2 | F |  |  |  |  | X | X | X | X |  |  |  | X |
| w1610 |  | 2 | M |  |  |  |  | X | X |  |  |  |  |  | X |
|  |  |  |  |  |  |  |  |  |  |  |  |  |  |  |  |
| w1604 | 2018 | 3 | F | Mix6 | Mix6 |  |  |  |  | X | X |  |  |  |  |
| w1701 |  | 2 | F |  |  |  |  |  |  |  |  |  |  |  |  |
| w1709 |  | 2 | F |  |  |  |  |  |  |  |  |  |  |  |  |
| w1710 |  | 2 | F |  |  |  |  | X | X |  |  |  |  |  |  |
| **N = 28** |  | **2,4 ± 0,09** | **Sex ratio : 9M/26F** | **n =6** | **n = 6** | **n = 8** | **n = 8** | **n = 5** | **n = 5** | **n = 6** | **n = 6** | **n = 8** | **n = 8** | n = 5 | n = 13 |

A total of 28 bears were included in this study, and all were captured both in February during winter hibernation (W) and in June during summer active period (S). Among them 4 bears (w1104, w1105, w1110 and w1509) were included two consecutive years. Serum mixes were prepared as followed: for a given year, 50 µl of summer serum from each bear of the year was pooled to obtain the summer mix. In parallel, 50 µl of winter serum from the same bears was pooled to obtain the winter mix. A total of 6 summer and winter paired mixes were obtained. Plasma was collected at the two time points (S and W) from 8 individual animals. Muscle and adipose tissues collected at the two time points (S and W) from respectively 5 and 6 individual animals. Total RNAs were obtained from muscle and adipose tissues collected at the two time points (S and W). For the muscle tissue, RNAs were obtained from 8 individual animals, while for adipose tissue, RNAs were obtained from 5 bears in summer and 13 bears in winter (only 4 paired samples from individual animals). N: number of bears, n: number of samples.

| **Fatty acid** | **WBS** | **SBS** | **FC W/S** | **pvalue** | **Adjusted p value (BH)** |
| --- | --- | --- | --- | --- | --- |
| ***C14:0*** *(myristic acid)* | 0.41 ± 0.04 | 0.16 ± 0.01 | 2.57 | 4.31E-04 | **1.44E-03** |
| ***C14:1*** *(myristoleic acid)* | 0.08 ± 0.01 | 0.02 ± 0.00 | 4.80 | 8.86E-04 | **2.00E-03** |
| ***C15:0*** *(pentadecanoic acid)* | 0.07 ± 0.01 | 0.06 ± 0.01 | 1.12 | 1.68E-01 | 2.19E-01 |
| ***C16:0*** *(palmitic acid)* | 6.99 ± 0.52 | 2.49 ± 0.17 | 2.80 | 1.13E-04 | **8.63E-04** |
| ***C16:1 n-9*** *(hypogeic acid)* | 0.18 ± 0.02 | 0.17 ± 0.02 | 1.04 | 7.37E-01 | 7.37E-01 |
| ***C16:1 n-7*** *(palmitoleic acid)* | 0.67 ± 0.06 | 0.22 ± 0.03 | 2.98 | 2.12E-05 | **6.36E-04** |
| ***C18:0*** *(stearic acid)* | 3.79 ± 0.16 | 2.45 ± 0.14 | 1.54 | 3.91E-04 | **1.44E-03** |
| ***C18:1 n-9*** *(oleic acid)* | 7.99 ± 0.63 | 4.70 ± 0.31 | 1.70 | 9.35E-04 | **2.00E-03** |
| ***C18:1 n-7*** *(vaccenic acid)* | 0.89 ± 0.08 | 0.34 ± 0.03 | 2.62 | 3.81E-04 | **1.44E-03** |
| ***C18:2 n-6*** *(linoleic acid)* | 4.04 ± 0.33 | 2.38 ± 0.17 | 1.70 | 6.74E-04 | **1.84E-03** |
| ***C18:3 n-6*** *(gamma-linolenic acid)* | 0.05 ± 0.02 | 0.05 ± 0.01 | 1.10 | 7.05E-01 | 7.29E-01 |
| ***C18:3 n-3*** *(alpha-linolenic acid)* | 0.15 ± 0.02 | 0.30 ± 0.06 | 0.49 | 7.35E-02 | 1.05E-01 |
| ***C20:0*** *(arachidic acid)* | 0.12 ± 0.01 | 0.11 ± 0.00 | 1.07 | 3.00E-01 | 3.33E-01 |
| ***C20:1 n-9*** *(gondoic acid)* | 0.05 ± 0.01 | 0.04 ± 0.01 | 1.21 | 2.13E-01 | 2.56E-01 |
| ***C20:2 n-6*** *(eicosadienoic acid)* | 0.32 ± 0.07 | 0.17 ± 0.05 | 1.91 | 3.00E-01 | 3.33E-01 |
| ***C20:3 n-6*** *(dihomo-gamma-linolenic acid)* | 0.18 ± 0.01 | 0.09 ± 0.01 | 2.02 | 2.68E-02 | **4.23E-02** |
| ***C20:4 n-6*** *(arachidonic acid)* | 1.86 ± 0.18 | 1.70 ± 0.26 | 1.09 | 1.94E-01 | 2.43E-01 |
| ***C20:5 n-3*** *(eicosapentaenoic acid)* | 0.10 ± 0.02 | 0.38 ± 0.01 | 0.26 | 9.32E-03 | **1.64E-02** |
| ***C22:0*** *(behenic acid)* | 0.10 ± 0.01 | 0.06 ± 0.00 | 1.69 | 3.95E-03 | **7.90E-03** |
| ***C22:1 n-9*** *(erucic acid)* | 0.02 ± 0.00 | 0.02 ± 0.00 | 1.32 | 3.39E-01 | 3.63E-01 |
| ***C22:5 n-3*** *(docosapentaenoic acid)* | 0.43 ± 0.05 | 0.16 ± 0.02 | 2.72 | 8.56E-04 | **2.00E-03** |
| ***C22:6 n-3*** *(docosahexaenoic acid)* | 0.32 ± 0.07 | 0.08 ± 0.02 | 3.81 | 7.03E-03 | **1.32E-02** |
| ***C24:0*** *(lignoceric acid)* | 0.11 ± 0.03 | 0.08 ± 0.03 | 1.47 | 7.37E-02 | 1.05E-01 |
| ***C24:1 n-9*** *(nervonic acid)* | 0.21 ± 0.03 | 0.09 ± 0.02 | 2.48 | 1.15E-02 | **1.92E-02** |
| **TOTAL** | **28.82 ± 1.71**  **7 1.46** | **15.99 ± 1.09** | **1.80** | 1.01E-04 | **8.63E-04** |
| *SFA* | 11.59 ± 0.70 | 5.42 ± 0.31 | 2.14 | 1.15E-04 | **8.63E-04** |
| *MUFA* | 10.09 ± 0.71 | 5.59 ± 0.33 | 1.80 | 3.36E-04 | **1.44E-03** |
| *PUFA* | 7.14 ± 0.63 | 4.98 ± 0.57 | 1.43 | 5.21E-04 | **1.56E-03** |
| *n-3 PUFA* | 0.94 ± 0.12 | 0.73 ± 0.14 | 1.30 | 8.19E-02 | 1.12E-01 |
| *n-6 PUFA* | 6.20 ± 0.51 | 4.25 ± 0.45 | 1.46 | 2.09E-04 | **1.25E-03** |

**Supplementary Table S2: Serum fatty acid concentrations (mmol/L) in winter hibernating (WBS) and summer active (SBS) bears.**

Lipidomic analysis were performed in bear serum as described in Material and Methods. Data are expressed in concentrations (mmol/L) of individual fatty acids. Data are represented as mean ± SEM of separate extractions and quantifications from the twelve bear serum mixes. FC W/S : fold change from winter / summer ratios, MUFA : monounsaturated fatty acids, PUFA : polyunsaturated fatty acids, SBS : summer bear serum, SFA : saturated fatty acids, WBS : winter bear serum. Paired Student t-test was used for season comparison and generation of p values. The Benjamini-Hochberg (BH) correction for multiple comparison was used to obtain the adjusted p values (below 0.05 in bold).

| **Fatty acid** | **WBS** | **SBS** | **FC W/S** | **p value** | **Adjusted p value (BH)** |
| --- | --- | --- | --- | --- | --- |
| ***C14:0*** *(myristic acid)* | 1.38 ± 0.07 | 1.00 ± 0.09 | 1.38 | 8.82E-03 | **1.76E-02** |
| ***C14:1*** *(myristoleic acid)* | 0.27 ± 0.02 | 0.11 ± 0.02 | 2.60 | 1.15E-03 | **4.93E-03** |
| ***C15:0*** *(pentadecanoic acid)* | 0.24 ± 0.02 | 0.39 ± 0.02 | 0.62 | 3.40E-03 | **8.50E-03** |
| ***C16:0*** *(palmitic acid)* | 24.18 ± 0.51 | 15.64 ± 0.45 | 1.55 | 1.91E-05 | **2.87E-04** |
| ***C16:1 n-9*** *(hypogeic acid)* | 0.62 ± 0.06 | 1.08 ± 0.06 | 0.57 | 2.56E-03 | **6.98E-03** |
| ***C16:1 n-7*** *(palmitoleic acid)* | 2.32 ± 0.13 | 1.42 ± 0.18 | 1.63 | 1.24E-04 | **1.24E-03** |
| ***C18:0*** *(stearic acid)* | 13.22 ± 0.28 | 15.41 ± 0.45 | 0.86 | 1.71E-03 | **5.70E-03** |
| ***C18:1 n-9*** *(oleic acid)* | 27.72 ± 1.47 | 29.52 ± 1.33 | 0.94 | 1.47E-01 | 1.84E-01 |
| ***C18:1 n-7*** *(vaccenic acid)* | 3.12 ± 0.31 | 2.19 ± 0.27 | 1.42 | 8.47E-03 | **1.76E-02** |
| ***C18:2 n-6*** *(linoleic acid)* | 14.08 ± 0.94 | 14.89 ± 0.44 | 0.95 | 3.66E-01 | 4.07E-01 |
| ***C18:3 n-6*** *(gamma-linolenic acid)* | 0.18 ± 0.05 | 0.29 ± 0.03 | 0.61 | 2.40E-02 | **3.79E-02** |
| ***C18:3 n-3*** *(alpha-linolenic acid)* | 0.50 ± 0.05 | 1.85 ± 0.38 | 0.27 | 1.47E-02 | **2.76E-02** |
| ***C20:0*** *(arachidic acid)* | 0.42 ± 0.02 | 0.71 ± 0.02 | 0.59 | 5.30E-06 | **1.59E-04** |
| ***C20:1 n-9*** *(gondoic acid)* | 0.19 ± 0.03 | 0.28 ± 0.05 | 0.67 | 1.03E-01 | 1.34E-01 |
| ***C20:2 n-6*** *(eicosadienoic acid)* | 1.05 ± 0.26 | 0.91 ± 0.14 | 1.15 | 7.85E-01 | 8.12E-01 |
| ***C20:3 n-6*** *(dihomo-gamma-linolenic acid)* | 0.67 ± 0.04 | 0.63 ± 0.04 | 1.07 | 5.31E-01 | 5.69E-01 |
| ***C20:4 n-6*** *(arachidonic acid)* | 6.41 ± 0.37 | 10.36 ± 0.81 | 0.62 | 9.45E-04 | **4.73E-03** |
| ***C20:5 n-3*** *(eicosapentaenoic acid)* | 0.31 ± 0.03 | 2.14 ± 0.16 | 0.15 | 1.65E-02 | **2.91E-02** |
| ***C22:0*** *(behenic acid)* | 0.35 ± 0.03 | 0.38 ± 0.04 | 0.92 | 9,17E-02 | 1.31E-01 |
| ***C22:1 n-9*** *(erucic acid)* | 0.08 ± 0.01 | 0.10 ± 0.00 | 0.78 | 2.92E-01 | 3.37E-01 |
| ***C22:5 n-3*** *(docosapentaenoic acid)* | 1.47 ± 0.08 | 0.97 ± 0.06 | 1.51 | 8.96E-04 | **4.73E-03** |
| ***C22:6 n-3*** *(docosahexaenoic acid)* | 1.11 ± 0.22 | 0.51 ± 0.09 | 2.17 | 2.17E-02 | **3.62E-02** |
| ***C24:0*** *(lignoceric acid)* | 0.41 ± 0.12 | 0.54 ± 0.21 | 0.77 | 2.89E-01 | 3.37E-01 |
| ***C24:1 n-9*** *(nervonic acid)* | 0.76 ± 0.13 | 0.56 ± 0.12 | 1.35 | 1.01E-01 | 1.34E-01 |
| *SFA* | 40.21 ± 0.46 | 34.08 ± 1.14 | 1.18 | 9.31E-04 | **4.73E-03** |
| *MUFA* | 35.04 ± 1.52 | 35.22 ± 1.57 | 0.99 | 8.75E-01 | 8.75E-01 |
| *PUFA* | 24.75 ± 1.42 | 30.70 ± 1.59 | 0.81 | 1.62E-03 | **5.70E-03** |
| *n-3 PUFA* | 3.23 ± 0.29 | 4.40 ± 0.66 | 0.73 | 5.97E-02 | 8.96E-02 |
| *n-6 PUFA* | 21.52 ± 1.17 | 26.30 ± 1.02 | 0.82 | 2.20E-03 | **6.60E-03** |
| *DHA/AA* | 0.17 ± 0.03 | 0.05 ± 0.01 | 3.44 | 3.80E-03 | **8.77E-03** |

**Supplementary Table S3: Serum fatty acid relative proportions (mol %) in winter hibernating (WBS) and summer active (SBS) bears.**

Lipidomic analysis were performed in bear serum as described in Material and Methods. Data are expressed in molar percentage (mol %) of total circulating lipids, reflecting proportions of individual fatty acids. Data are represented as mean ± SEM of separate extractions and quantifications from the twelve bear serum mixes. FC W/S : fold change from winter / summer ratios, MUFA : monounsaturated fatty acids, PUFA : polyunsaturated fatty acids, SBS : summer bear serum, SFA : saturated fatty acids, WBS : winter bear serum. Paired Student t-test was used for season comparison and generation of p values. The Benjamini-Hochberg (BH) correction for multiple comparison was used to obtain the adjusted p values (below 0.05 in bold).

**Supplementary Table S4 : List of primers used for RT-qPCR**

| Gene |  | Primer |
| --- | --- | --- |
| Ua.TBP | Forward | 5’- AGACCATTGCACTTCGTGCC -3’ |
|  | Reverse | 5’- CCTGTGCACACCATTTTCCC -3’ |
| Ua.CNR1 | Forward | 5’- GTTGCCGAGGGAGCTTCTCC -3’ |
|  | Reverse | 5’- TGTCGGCAAGGCCATCTAGG -3’ |
| Ua.CNR2 | Forward | 5’- AACTCAACATGTCGGCAGCC -3’ |
|  | Reverse | 5’- CCATCACCCAGCGTCTCTCC -3’ |
| Ua.NAPEPLD | Forward | 5’- TTTGACCTTGCAGCTATTCC -3’ |
|  | Reverse | 5’- GATTCTCCATGCTTCAAGAC -3’ |
| Ua.FAAH | Forward | 5’- AAGCAACATACCCCATGCTC -3’ |
|  | Reverse | 5’- GGTCCACGAAATCACCTTTG -3’ |
| Ua.DAGLA | Forward | 5’- CACGTGGTCCACAACCACCC -3’ |
|  | Reverse | 5’- AGGTGCTCATGCAGCATGGC -3’ |
| Ua.DAGLB | Forward | 5’- GTGCTGCTGTGTCGGGAAGG -3’ |
|  | Reverse | 5’- GGCACCAGATCTGTGTCTGA -3’ |
| Ua.MGLL | Forward | 5’- GCGTGCTCTCTCGGAATAAG -3’ |
|  | Reverse | 5’- TTGCCGAAGCACACCTTCAG -3’ |

**Supplementary Table S5 : Endocannabinoids (eCBs) and mRNA quantification in plasma and tissues in winter hibernating (W) and summer active (S) bears.**

| ***eCBs in plasma (ng/mL)*** | |  |  |  |
| --- | --- | --- | --- | --- |
|  | **WBP** | **SBP** | **FC W/S** | **p value** |
| *AEA* | 0.66 ± 0,06 | 1,06 ± 0,14 | 0,63 | **1,14E-02** |
| *2-AG* | 6,20 ± 0,51 | 6,67 ± 0,74 | 0,93 | 6,10E-01 |
| *OEA* | 24,02 ± 2,06 | 7,35 ± 1,09 | 3,27 | **3,18E-04** |
|  |  |  |  |  |
| ***eCBs in muscle tissue (pg/mg tissue)*** | | |  |  |
|  | **WBM** | **SBM** | **FC W/S** | **p value** |
| *AEA* | 0,58 ± 0,07 | 1,56 ± 0,38 | 0,37 | 6,45E-02 |
| *2-AG* | 28,05 ± 1,20 | 45,46 ± 2,67 | 0,62 | **4,87E-03** |
| *OEA* | 15,18 ± 1,06 | 16,55 ± 2,97 | 0,92 | 5,89E-01 |
|  |  |  |  |  |
| ***eCBs in adipose tissue (pg/mg tissue)*** | | |  |  |
|  | **WBA** | **SBA** | **FC W/S** | **p value** |
| *AEA* | 0,70 ± 0,06 | 1,58 ± 0,36 | 0,44 | 6,92E-02 |
| *2-AG* | 14,56 ± 0,90 | 129,89 ± 33,09 | 0,11 | **1,69E-02** |
| *OEA* | 12,87 ± 1,39 | 12,63 ± 3,29 | 1,02 | 9,40E-01 |
|  |  |  |  |  |
| ***Gene expression in muscle tissue (normalized to TBP)*** | | | |  |
|  | **WBM** | **SBM** | **FC W/S** | **p value** |
| *CNR1* | 0.63 ± 0.15 | 1.00 ± 0.15 | 0,63 | **3,40E-02** |
| *CNR2* | 1.07 ± 0.23 | 1.00 ± 0.15 | 1,07 | 7,98E-01 |
| *FAAH* | 2.32 ± 0.45 | 1.00 ± 0.13 | 2,32 | **1,23E-02** |
| *MGLL* | 1.11 ± 0.13 | 1.00 ± 0.10 | 1,11 | 5,09E-01 |
| *DAGLA* | 0.41 ± 0.06 | 1.00 ± 0.23 | 0,45 | **2,72E-02** |
| *DAGLB* | 1.54 ± 0.14 | 1.00 ± 0.14 | 1,54 | **1,07E-02** |
| *NAPEPLD* | 0.77 ± 0.06 | 1.00 ± 0.11 | 0,77 | 7,03E-02 |
|  |  |  |  |  |
| ***Gene expression in adipose tissue (normalized to TBP)*** | | | |  |
|  | **WBA** | **SBA** | **FC W/S** | **p value** |
| *CNR1* | 1.65 ± 0.39 | 1.00 ± 0.31 | 1,65 | 3,39E-01 |
| *CNR2* | 0.42 ± 0.05 | 1.00 ± 0.23 | 0,42 | **2,24E-03** |
| *FAAH* | 0.75 ± 0.10 | 1.00 ± 0.25 | 0,75 | 2,85E-01 |
| *MGLL* | 1.40 ± 0.15 | 1.00 ± 0.21 | 1,40 | 1,78E-01 |
| *DAGLA* | 0.94 ± 0.11 | 1.00 ± 0.24 | 0,94 | 8,12E-01 |
| *DAGLB* | 1.43 ± 0.06 | 1.00 ± 0.09 | 1,44 | **2,08E-03** |
| *NAPEPLD* | 0.75 ± 0.06 | 1.00 ± 0.07 | 0,75 | **3,25E-02** |

Quantification were performed as described in the Methods section. Data are expressed as Mean ± SEM. For gene expression, mRNA levels were normalized to TBP expression then expressed related to the summer values. Season differences were assessed using the Student paired t-test, except for gene expression in adipose tissue where unpaired t-test were used, and p values are reported.
